# Assessing lymphatic route of CSF outflow and peripheral lymphatic contractile activity during head‐down tilt using near‐infrared fluorescence imaging

**DOI:** 10.14814/phy2.14375

**Published:** 2020-02-25

**Authors:** John C. Rasmussen, Sunkuk Kwon, Amanda Pinal, Alexander Bareis, Fred C. Velasquez, Christopher F. Janssen, John R. Morrow, Caroline E. Fife, Ron J. Karni, Eva M. Sevick‐Muraca

**Affiliations:** ^1^ Center for Molecular Imaging The Brown Foundation Institute of Molecular Medicine at The University of Texas Health Science Center at Houston Houston TX USA; ^2^ Center for Laboratory Animal Medicine and Care The University of Texas Health Science Center at Houston Houston TX USA; ^3^ Department of Geriatrics Baylor College of Medicine Houston TX USA; ^4^ The Wound Care Clinic CHI St. Luke’s Health The Woodlands Hospital The Woodlands TX USA; ^5^ Department of Otorhinolaryngology The University of Texas Health Science Center at Houston Houston TX USA

**Keywords:** cerebrospinal fluid, CSF outflow, lymphatics, microgravity, near‐infrared fluorescence imaging, neurodegeneration

## Abstract

Evidence overwhelmingly suggests that the lymphatics play a critical role in the clearance of cerebrospinal fluid (CSF) from the cranial space. Impairment of CSF outflow into the lymphatics is associated with a number of pathological conditions including spaceflight‐associated neuro‐ocular syndrome (SANS), a problem that limits long‐duration spaceflight. We used near‐infrared fluorescence lymphatic imaging (NIRFLI) to dynamically visualize the deep lymphatic drainage pathways shared by CSF outflow and disrupted during head‐down tilt (HDT), a method used to mimic the cephalad fluid shift that occurs in microgravity. After validating CSF clearance into the lymph nodes of the neck in swine, a pilot study was conducted in human volunteers to evaluate the effect of gravity on the flow of lymph through these deep cervical lymphatics. Injected into the palatine tonsils, ICG was imaged draining into deep jugular lymphatic vessels and subsequent cervical lymph nodes. NIRFLI was performed under HDT, sitting, and supine positions. NIRFLI shows that lymphatic drainage through pathways shared by CSF outflow are dependent upon gravity and are impaired under short‐term HDT. In addition, lymphatic contractile rates were evaluated from NIRFLI following intradermal ICG injections of the lower extremities. Lymphatic contractile activity in the legs was slowed in the gravity neutral, supine position, but increased under the influence of gravity regardless of whether its force direction opposed (sitting) or favored (HDT) lymphatic flow toward the heart. These studies evidence the role of a lymphatic contribution in SANS.

## Graphical abstract

Near‐infrared fluorescence lymphatic imaging (NIRFLI) was used to dynamically visualize the deep lymphatic drainage pathways shared by CSF outflow and disrupted during head‐down tilt (HDT), a method used to mimic the cephalad fluid shift that occurs in microgravity. After validating CSF clearance into the lymph nodes of the neck in swine, a pilot study was conducted in human volunteers to evaluate the effect of gravity on the flow of lymph through these deep cervical lymphatics. NIRFLI shows that lymphatic drainage through pathways shared by CSF outflow are dependent upon gravity and are impaired under short‐term HDT and that lymphatic contractile activity in the legs was slowed in the gravity neutral, supine position, but increased under the influence of gravity.

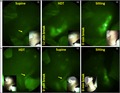

## 1 INTRODUCTION

Impaired CSF outflow into the lymphatics may play a role in aging (Ahn et al., 2019; Ma, Ineichen, Detmar, and Proulx 2017); in the etiology of neurodegenerative disease (Da Mesquita et al., 2018; Kwon et al., 2019; Patel et al., 2019); and contribute to spaceflight‐associated neuro‐ocular syndrome (SANS) formerly known as visual impairment and intracranial pressure (VIIP) syndrome (Leach et al., 1996; Parazynski et al., 1991). The syndrome manifests with an increase in intracranial and intraocular pressures (ICP and IOP) presumably resulting from microgravity‐induced headward fluid shift. SANS may represent a significant physiological barrier to long‐duration space flight. In addition to the intracranial and intraocular pressure, astronauts also report edema of the face and head, and a shrinking, or the functional opposite of edema, in the lower extremities.

Starling's principle, and later revisions of it (Levick and Michel, 2010), describe a balance of pressures, including hydrostatic and oncotic, that govern the movement, by osmosis, of water and permeable solutes, out of the circulatory system, specifically arterioles, capillaries, venules, and into the surrounding tissues. The function of the lymphatic system is to gather this excess water, called interstitial fluid, into lymphatic ducts, at which point the fluid is termed lymph, and to deliver it back into the blood circulatory system. The lymphatic ducts parallel the veins, becoming the thoracic duct, which finally delivers the collected lymph from the entire body into the vena cava near the heart. Because most of the body is below the level of the heart, the lymphatic ducts are equipped with many one‐way valves and contractile walls, forming pumping units called lymphangions or “lymph hearts,” which can move lymph against gravity. Lymph nodes are strategically placed “filters” positioned along the lymph ducts and are used by the immune system to sample the fluid returning from the body for surveillance of infection or foreign materials. The clinical condition of edema or lymphedema is a gelatinous swelling of tissue due to accumulated water in the interstitial space. Edema or lymphedema results when interstitial fluid is produced faster than the lymphatics can remove it. The pressures that are summed in Starling's equation include the mean arterial pressure, generated by the beating heart; the oncotic pressure, best described as the osmotic pressure generated by immobile solutes such as albumin in the blood vessels and glycoproteins in the tissue; and, of specific interest to this project, the hydrostatic pressures generated by the pull of gravity on blood. Similarly, we are interested in the effect of hydrostatic pressure on the movement of lymph within the lymphatics.

Under terrestrial conditions in an upright human, the lymphangions in the lower extremities actively pump lymph against gravity to lift the lymph to the level of the heart. Because the head and neck are above the level of the heart, lymphatic drainage from these regions is assisted by gravity. Since the work of Schwalbe in 1869 (Schwalbe, 1869), it has been known that CSF from the subarachnoid space drains into the major lymphatics of the head and neck (Foldi et al., 1968) and therefore should be aided by gravity. However, the extent of drainage through this pathway and how drainage through this pathway can be impacted by disease and by external factors has received little attention, even though humans produce as much as 600–700 cc of CSF each day (Brinker, Stopa, Morrison, & Klinge, 2014).

Most textbooks state CSF absorption into the venous arachnoid villi represents the major clearance mechanism, but recent revisions in Starling's law make significant venous reabsorption improbable (Levick & Michel, 2010). Indeed, intracranial outflow of CSF occurs predominantly through the cribriform as well as basal and meningeal lymphatics (Ahn et al., 2019; Louveau et al., 2015), which ultimately drain into the jugular lymphatic channels and into the deep cervical LNs. Intracranially, CSF from the subarachnoid space drains into perivascular (Virchow–Robin) spaces, from where it may exchange with the interstitial fluid before emptying into the perivenous compartments and into the deep jugular and cervical lymphatics (Kwon, Janssen, Velasquez, & Sevick‐Muraca, 2017; Ma et al., 2017). During spaceflight, there is no gravity to aid lymph drainage of the head and neck, nor for lymphangions in the lower extremities to pump against. The result may be congestion of the cervical lymphatics, impaired CSF outflow, and increased ICP. Through this mechanism, the facial edema and headache commonly observed in astronauts may also be associated with the long‐term effects of optic disc edema and optic nerve sheath distension (Nelson, Mulugeta, & Myers, 2014), and with the development of white matter hyperintensities (WMHs) (Alperin, Bagci, & Lee, 2017) similar to those observed in aging populations. Given that disorders of CSF outflow can lead to severe central nervous system (CNS) diseases such as hydrocephalus (Johnston & Papaiconomou, 2002) and may play a significant role in neurodegenerative, neurovascular, and neuroinflammatory diseases (Simon & Iliff, 2016), as well as psychiatric illnesses (Najjar, Pearlman, Alper, Najjar, & Devinsky, 2013), microgravity‐induced changes in CSF outflow could be consequential. The lack of a noninvasive method to image changes in CSF outflow hinders an understanding of how it may contribute to these conditions. Herein we develop a simple, minimally invasive technique for clinically imaging CSF outflow into the lymphatics that may help elucidate both normal CSF physiology and the pathophysiologic impact of microgravity.

Our work focused upon imaging the CSF drainage pathway using intrathecal injection of indocyanine green (ICG) followed by near‐infrared fluorescence lymphatic imaging (NIRFLI). Unfortunately, intrathecal injection of ICG (as currently formulated) is contraindicated in humans because the low oncotic pressure of CSF creates the potential for ICG precipitation and neurotoxicity (Dietz & Jaffe, 2003; Jackson, 2005; Toczylowska et al., 2014). As a result, we first used a swine model to demonstrate drainage pathways into the lymphatic vasculature. While we were able to validate the important anatomical and physiological drainage pattern in swine, quadrupeds are inadequate models with which to observe the gravity‐driven CSF outflow of human bipeds. Fortunately, intracranial CSF outflow shares a pathway with the deep lymphatic channels running adjacent to the human jugular vein that drain the palatine tonsils. Consequently, NIRFLI could be used to visualize the passive drainage of deep lymphatics in human volunteers by injecting trace doses of ICG into the palatine tonsil. Using this minimally invasive technique, we observed alterations in cervical lymph flow under conditions of head‐down tilt (HDT), or the Trendelenberg position, which is an accepted model of the cephalad fluid shift experienced in space (Watenpaugh, 2016). We also imaged the changes in contractile activity of lymphangions in the lower extremities when subjects were placed in gravity neutral (supine), gravity aided (HDT), and gravity opposing (sitting) positions. Our results show, for the first time in humans, that lymphatic function and drainage are gravity dependent and that microgravity could contribute to the etiology of SANS. The opportunity to evaluate the way in which CSF outflow contributes to the pathophysiology of SANS represents a major step forward in a heretofore mysterious problem which could limit space exploration. The method of imaging CSF outflow described herein has implications for research in oncology, psychiatry, neurodegenerative, and neuroinflammatory diseases (Najjar et al., 2013). The implications of our study argue for the development of new, ICG formulations safe for intrathecal injection in humans, and new methods for intrathecal administration of ICG.

## 2 MATERIALS AND METHODS

### 2.1 Animal studies

Experiments were conducted on three female Yorkshire swine (4.5 months old, 26–50 kg, MDA Bastrop) as approved by and in accordance with the guidelines the University of Texas Health Science Center at Houston's Animal Welfare Committee. The animals were fasted for 12 hr prior to initiating anesthesia. The animals were anesthetized initially using an intramuscular injection of tiletamine/zolazepam and then maintained with isoflurane throughout the procedure to minimize distress. Throughout the procedure, the vitals were monitored using a multiparameter monitor and the animals were ventilated, intravenous fluids were provided via a catheter, and body temperature was maintained at 98ºF using a warming blanket and air blower. The animals’ backs were shaved to limit the impact of hair during imaging.

The lower back region was disinfected using betadine and a small incision (~2 cm) made between the L5 and L6 vertebrae. Using spinal needles (McKesson, #4631V2, 22g × 3 ½ inch), intrathecal administration of 5–10 ml of 0.25 mg/ml solution of ICG in saline was made between the L5‐6 of anesthetized swine. A tail flick response was observed as indication of correct position of the needle in the intradural space and near‐infrared fluorescence (NIRF) imaging was conducted for 1–3 hr immediately after intrathecal injection of ICG. The purpose of NIRF imaging was to noninvasively visualize ICG transit through the spinal canal to the cisterna magna, around brain parenchyma, out through the olfactory lymphatics, and into cervical LNs. ICG administration appeared not to impart any neurological defects in the animals. After imaging two animals were allowed to fully recover and were provided their normal rations and daily enrichment. The following day, the animals were again sedated using tiletamine/zolazepam and then, after deeply sedated, euthanized with pentobarbital sodium IV. The third animal was euthanized immediately following imaging (~1.5 hr post injection). Following euthanasia, tissue collection was performed under fluorescence guidance using the residual ICG to assess its trafficking through the above‐named structures.

### 2.2 Human subjects

Three female and two male healthy volunteers (average 38 years of age, range 28–65) were recruited for this pilot study. To participate in the study, volunteers had to (a) be at least 18 years of age, (b) be able to lie on their backs for up to 5 hr, (c) not be taking prescription medication (with the exception of birth control); (d) be nonsmokers; (e) have a body mass index between 19 and 30 kg/m^2^; (f) have no known allergy to iodine; (g) no history of increased ICP, neurological, lymphatic, or cardiovascular disease; and (h) if female, not be pregnant or breast‐feeding and agree to use a medically accepted method of contraception for a period of one month following the study. Subjects participated in two (2) imaging sessions separated by 8 to 18 days (Mean = 12.2 days). The study protocol was conducted under the approved Institutional Review Board at the University of Texas Health Science Center at Houston and the Food and Drug Administration under combinational investigational new drug application (IND) 106,765 for off‐label administration and use of indocyanine green (ICG).

**Figure 1:**
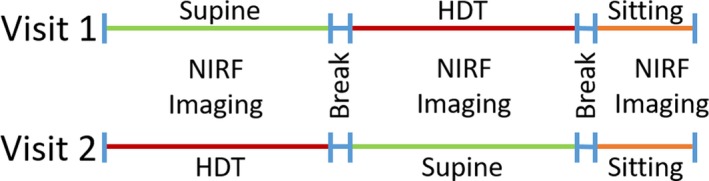
Timeline depicting the supine, HDT, and sitting positions of the study subjects in Visit 1 and Visit 2

After informed consent, a total of eight intradermal injections of 25 µg of ICG in 0.1 ml saline were administered bilaterally with an injection on each medial ankle and two on the dorsum of each foot using 31‐guage insulin needles (BD, 328438). The remaining two injections were administered bilaterally into the palatine tonsils, using a 25‐gauge spinal needle (Braun, S25475) to reach the back of the mouth, following the optional application of oral lidocaine spray. The intradermal injections were covered with a round bandage and black vinyl tape to prevent oversaturation of the imaging system. Immediately after all injections, the subject was placed in either a supine position for approximately 1.5 hr or in a HDT position (between −12° and −18°) for as long as they could tolerate up to 3.5 hr, (range was 1 hr 13 m to 3 hr 31 m). During these times, NIRFLI was conducted to assess contractile activity in both left and right lower extremities and to follow ICG drainage into the cervical LNs. Subjects were allowed to take a brief 5–10 min break before being placed in the alternate position, whether HDT or supine. Finally, after a brief break, subjects were imaged sitting upright for approximately 30 min. During these times, NIRFLI was conducted to assess contractile activity in both lower extremities and ICG drainage into the left and right cervical LNs. The images provided real‐time visualization of the lymphatic architecture and, from collected images, allowed computation of contractile function and analysis of fluorescence intensity in standard international units in draining cervical LNs. In the second visit, the same methodology was used except the order of supine and HDT positions was changed. While ICG cleared from the lymphatics, retention of trace ICG in the prior intradermal injection sites remained detectable in NIRF images at Visit 2 and allowed for reinjection at the same sites for evaluation of the same lymphatic vessels. Figure 1 is a schematic of the protocol used. During the breaks, subjects stood up and moved around, often taking a bathroom break.

### 2.3 NIRF lymphatic imaging

For both swine and human imaging, NIRFLI was conducted using the custom, variable focus, and variable field of view (350–1,900 cm^2^), imaging system as described previously (Rasmussen, Tan, et al., 2017; Rasmussen, Zvavanjanja, Aldrich, Greives, & Sevick‐Muraca, 2017) and consisting of a Gen III intensified, scientific complementary metal–oxide–semiconductor (IsCMOS) camera, 830 nm bandpass filters, and a 28 mm Nikon lens. To acquire images, the surface of the skin was illuminated with a diffused 785 nm excitation light of <1.9 mW/cm^2^, and emitted fluorescence was collected with 0.2 s exposure times at approximately 3 frames/sec. Imaging alternated between the face and neck and the legs, generally in 20 min segments. Because the system employs a near‐infrared image intensifier that amplifies the collected fluorescence and integrates the amplified signal on a sCMOS chip, the system allows sensitivity for detecting LNs and lymphatic vessels as deep as 3–4 cm beneath the tissue surface (Sevick‐Muraca et al., 2008). While the IsCMOS system has submillimeter resolution, (<300 µm) as previously measured using a USAF 1951 standard resolution target (Zhu, Rasmussen, Litorja, & Sevick‐Muraca, 2016), because tissue scatters light, the effective spatial resolution of the technique decreases as the depth of the fluorescent lymphatic drainage pathways increases, resulting in deeper lymphatic vessels appearing to have enlarged diameters.

### 2.4 NIRFLI analysis of contractile activity in lower extremities

From the acquired images in humans, contractile activity was assessed in both right and left legs over periods of 20 min each during the HDT and supine positions and 10 min in the sitting position. As previously described (Tan et al., 2011), the number of “pulses” or boluses of propelled ICG‐laden lymph and their transit velocities were quantified as they transited through the lymphatic vessels. Briefly, the velocity was determined by (a) drawing regions of interest (ROI) along each lymphatic vessel, initiating approximately 1–3 cm above the medial ankle injection sites; (b) plotting the fluorescence intensity at two ROIs, typically near the beginning and end of the series of ROIs; and (c) extracting the time points at which the maximum signal from each bolus passed through the each ROI. The velocity was then calculated by dividing the distance between the two ROIs by the travel time between the regions. The average velocity of boluses in all observed vessels was computed for each position at each visit. The total number of pulses observed in each vessel was divided by the total imaging time over which the pulses were observed to compute the rate of propulsion for each vessel. The average propulsion rate in all observed vessels was calculated for each position at each visit. Differences in lymphatic contractile activities as a function of position were assessed through paired, two‐tailed *t*‐tests with significance denoted at the level of *p* < .05.

### 2.5 NIRFLI assessment of lymphatic drainage in pathway of CSF outflow

For assessment of lymphatic drainage into the cervical lymph nodes, a traceable, NIRF stable working standard (Zhu et al., 2016) of known and constant fluorescent characteristic (with units of radiance (pW/cm^2^/sr) at the emission wavelength/ irradiance (pW/cm^2^) at excitation wavelength) was placed in the field of view. The fluorescence intensity emanating from the right and left cervical LNs was reported relative to the working standard whose fluorescence output is equivalent to ~ 18 nM of ICG (Zhu, Rasmussen, & Sevick‐Muraca, 2014), allowing comparison of results across study visits and study subjects. The increase in the relative fluorescence as a function of time in the cervical LNs was used as a measure of drainage in the lymphatic pathway of CSF outflow. Because the use of the working standard was optimized in the first two study subjects, CSF outflow results are presented only for the last three subjects.

## 3 RESULTS

### 3.1 CSF drainage following intrathecal injection in large animal model

**Figure 2:**
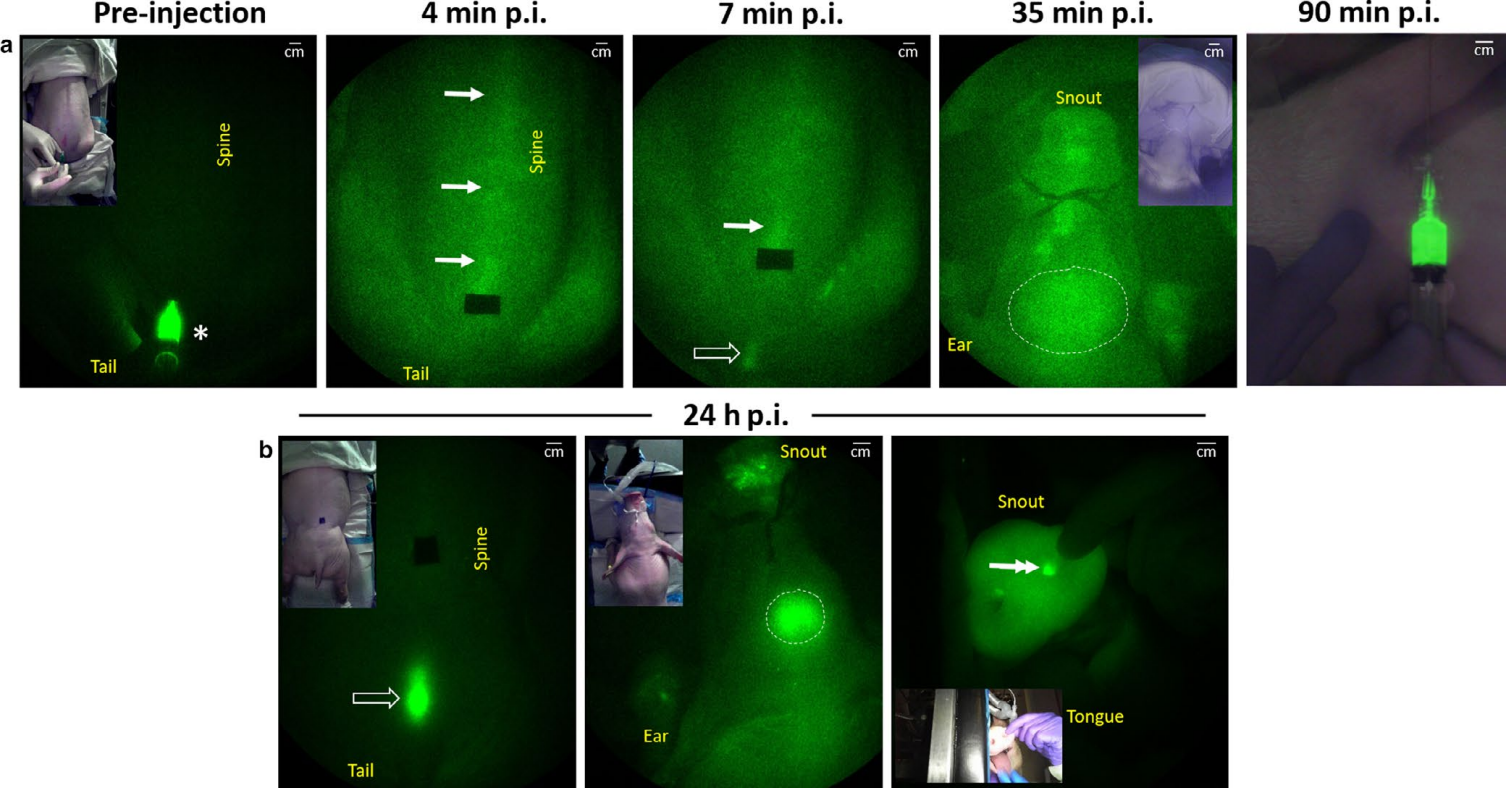
NIRF images with inset color images acquired (a) pre‐injection and at 4 min, 7 min, 35 min, 90 min and (b) 24 hr after intrathecal injection of ICG. (a) The pre‐injection image shows the syringe (asterisk), injection site, and spine immediately before ICG administration; the 4 and 7 min images show the faint but visible fluorescent signal along the spine (arrows) proximal to the injection site (black vinyl tape) and distal to the injection site at the sacral‐caudal junction (open arrow); the 35 min image shows the fluorescent signal emanating from the brain (white dotted circle); the 90 min is an overlay of the fluorescence and color images of a CSF‐filled syringe obtained from the cisterna magna. (b) 24 hr p.i. images show the fluorescence signal at the sacral‐caudal junction (open arrow) and through the cranium (white dotted circle), as well as at the nares (double arrow). NIRF images are presented in pseudo color and have been adjusted for brightness and contrast to enhance visualization of the full 16‐bit image depth information. The injection site has been covered with black vinyl tape in (a) 4 min p.i., 7 min p.i., and the left image in (b)

**Figure 3:**
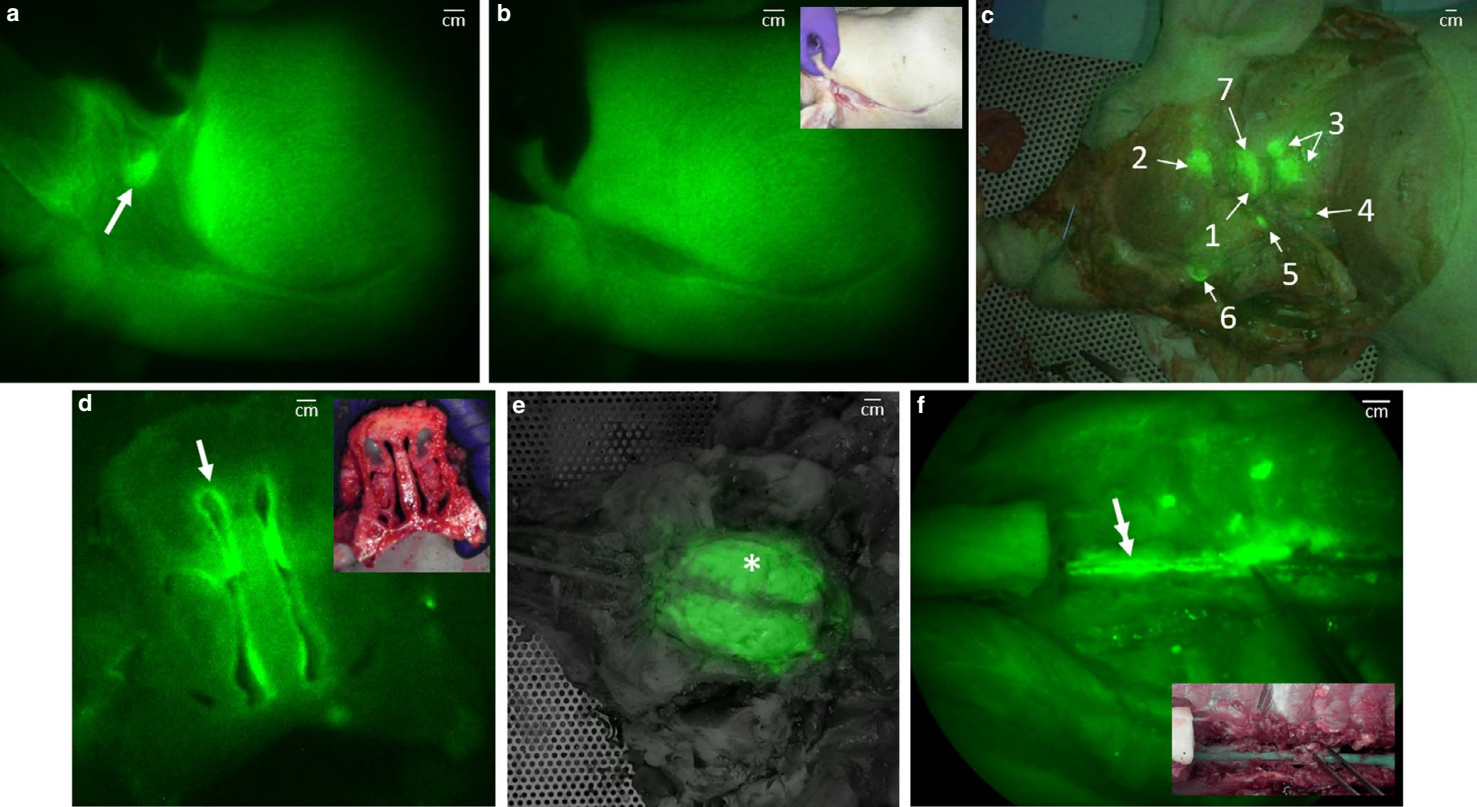
Post‐mortem NIRF images with inset color images of CSF drainage. (a‐b) The right lateral view around the neck showing a fluorescent parotid LN (arrow, a) without the skin and (b) covered with the skin. (c) Overlay of post‐mortem NIRF and color images showing medial cervical area denoting fluorescence in the following LNs (1) lateral retropharyngeal, (2) parotid, (3) dorsal superficial cervical, (4) ventral superficial cervical, (5) mandibular, (6) accessary mandibular, and (7) medial retropharyngeal LNs. (d‐f) Images show the fluorescence signal from the (d) nasal cavity (arrow), (e) excised brain (asterisk), and (f) spinal cord (double arrow). NIRF images are presented in pseudo color and have been adjusted for brightness and contrast to enhance visualization of the full 16‐bit image depth information

Minimally invasive NIRF imaging demonstrated ICG fluorescence along the spine (arrows at 4 and 7 min post injection (p.i.) in Figure 2a) and through the cranium (white dotted circle at 35 min p.i. in Figure 2a), indicative of CSF flow after intrathecal injection of ICG. CSF drainage after intrathecal injection was also confirmed by collecting CSF from the cisterna magna (90 min p.i. in Figure 2a). At 24 hr p.i. of ICG, strong NIRF signals were detected near the skin at the sacral‐caudal junction (open arrow in Figure 2b) and through the cranium (white circle in Figure 2b) as well as at the nares (double arrows in Figure 2b). However, no ante‐mortem signal could be seen from peripheral LNs in the neck region. Minimal to no ICG fluorescence was collected from the spine and peripheral LNs because (i) the spine of the swine is located deep (e.g., measured to be 9 and 4 cm from cervical and lumbar vertebrae to the skin, respectively) and (ii) the skin of the swine causes significant reflection and scatter of NIR light in comparison to humans. As shown in Figure 3a (arrow), a fluorescent parotid LN is clearly visualized after the swine were euthanized, 24 hr after ICG administration, and the skin and fatty tissues were removed. However, the fluorescent LN is not detectable (Figure 3b) when the skin and fatty tissues are replaced over the LN, primarily due to high tissue scattering/absorption and the resulting background signal. After further removal of skin, fat, and muscle, we visualized fluorescence in the lateral retropharyngeal, parotid, dorsal superficial cervical, ventral superficial cervical, mandibular, accessary mandibular, and medial retropharyngeal LNs (Figure 3c). Upon removal of the skin and fat, lymph nodes were already faintly visible in the neck of the animal euthanized 1.5 hr after injection.

**Figure 4:**
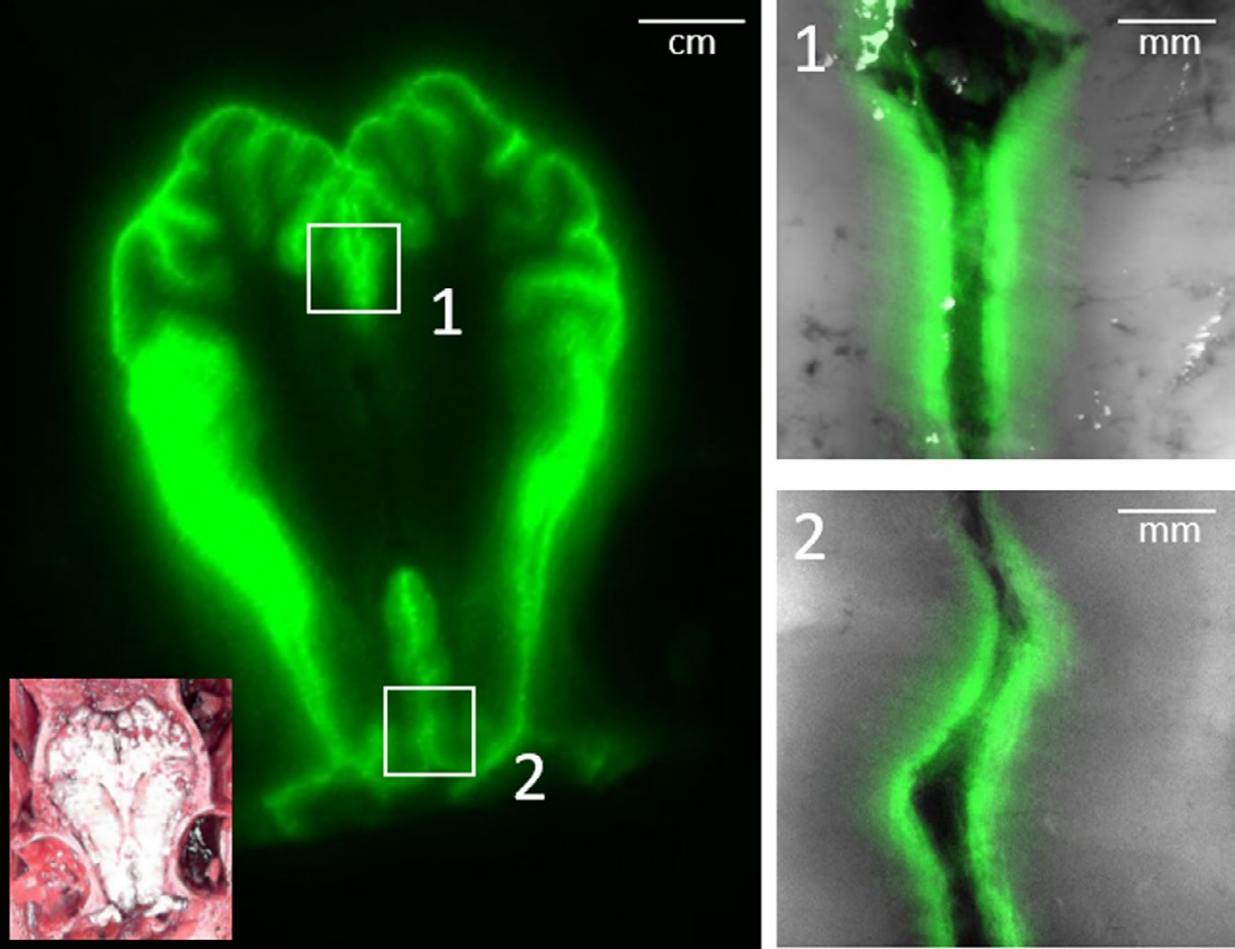
Cross‐sectional post‐mortem NIRF images of the brain showing Robin–Virchow spaces involved in the transit of CSF into the LNs with overlays of zoomed‐in NIRF and white light images of rectangles (1 and 2). Inset, color image of cross‐sectional brain. NIRF images are presented in pseudo color and have been adjusted for brightness and contrast to enhance visualization of the full 16‐bit image depth information

Gross examination showed ICG fluorescence in the submucosa outside the nasal meatus, suggesting CSF drainage to the nasal mucosal lymphatics through the cribriform plate (Figure 3d). We detected ICG fluorescence in excised brain (Figure 3e) and the spinal cord, which also showed green color (inset in Figure 3f). A fluorescent image of the cross section of the brain showed ICG filling of apparent perivascular (Robin–Virchow) spaces (Figure 4) as previously shown after intrathecal injection in mice (Kwon et al., 2017).

### 3.2 Lymphatic drainage into pathway of CSF drainage in human volunteers as a function of position

**Figure 5:**
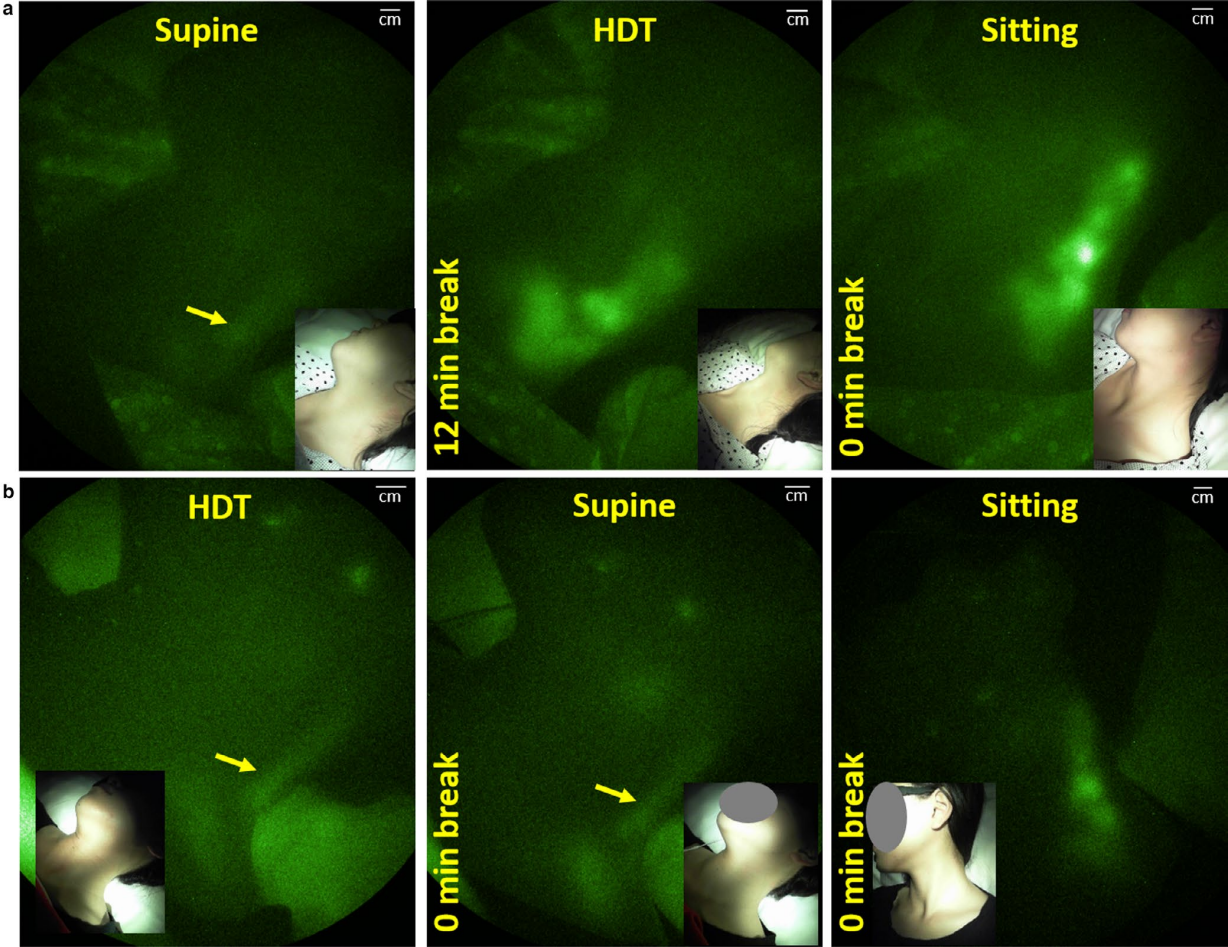
(a)‐(b) Typical fluorescence imaging of the right (a) and left (b) lateral neck and cervical views during HDT, supine, and sitting positions. NIRF images are presented in pseudo color and have been adjusted for brightness and contrast to enhance visualization of the full 16‐bit image depth information

**Figure 6:**
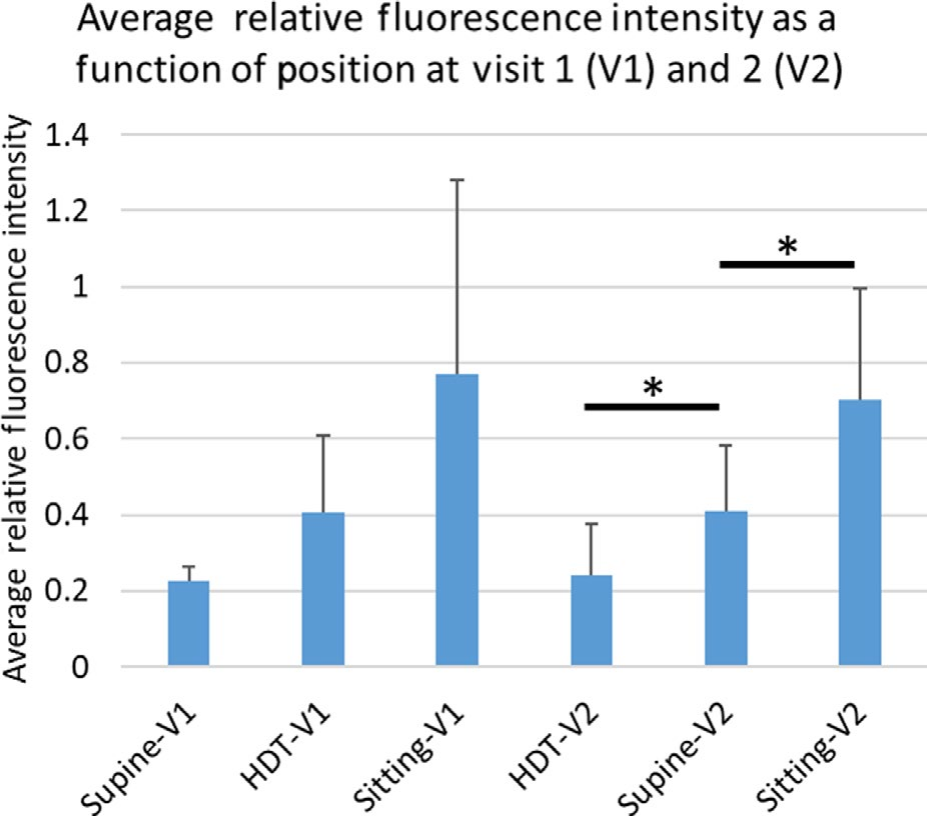
Plot of relative fluorescence (in terms of ICG concentration) as a function of position in Visits 1 and 2 for the last three subjects. *denotes *p* < .05

The palatine tonsil injections in the normal volunteers were well‐tolerated and no adverse events were associated with these off‐label mucosal or intradermal administrations. Most, but not all, bilateral tonsil injections resulted in deep lymphatic drainage. The inability to see deep lymphatic drainage in every case is presumably due to poor uptake of ICG into the lymphatics resulting from the inability to precisely control the depth of injection using the spinal needle. However, of 20 injections across all visits and all subjects, we deemed 18 injections successful as evidenced by lymphatic drainage to the cervical LNs. While subcutaneously injected lidocaine has been shown to temporarily reduce lymphatic contractility for 15 min in mice (Kwon & Sevick‐Muraca, 2016), there appeared no difference in the cervical drainage assessed over the hours of study time between the subjects who opted or did not opt for topical lidocaine. Figure 5a and 5b provide examples of imaged drainage through the deep lymphatic vessels into the cervical LNs in one of the subjects at Visit 1 following supine, HDT, and sitting positions, and then at Visit 2 following HDT, supine, and sitting positions. When gravity opposed normal drainage (under HDT), or in the absence of gravity‐driven drainage (in supine position), the fluorescence in the cervical LNs was less when compared to the sitting position as shown in Figure 5. Between Visits 1 and 2, there was no statistical difference between cervical LN intensities in the first position, whether supine or HDT. While drainage occurred during the break between supine to HDT positions (Visit 1), further drainage appeared to occur in some participants when placed in HDT as indicated by an increased LN intensity; however, no significant increase was observed across the population. When subjects were moved from HDT to supine (Visit 2) position, significant increases (*p* < .05, paired *t *test), in LN intensities occurred suggesting enhanced drainage in the gravity neutral position. Upon positioning to sitting posture, from HDT or supine positions, LN intensities tended to increase from the HDT position (Visit 1) and significantly increased (*p* < .05, paired *t *test), from the supine position (Visit 2). Figure 6 plots the relative fluorescence (in terms of ICG concentration) of the cervical LNs of those patients as a function of drainage in the supine (unaided by gravity), HDT (opposed to gravity), and sitting (in the direction of gravity) positions at Visit 1 and as a function of drainage in the HDT (opposed to gravity), supine (unaided by gravity), and sitting (in the direction of gravity) at Visit 2.

### 3.3 Lymphatic contractility in lower extremities in human volunteers as a function of position

**Figure 7:**
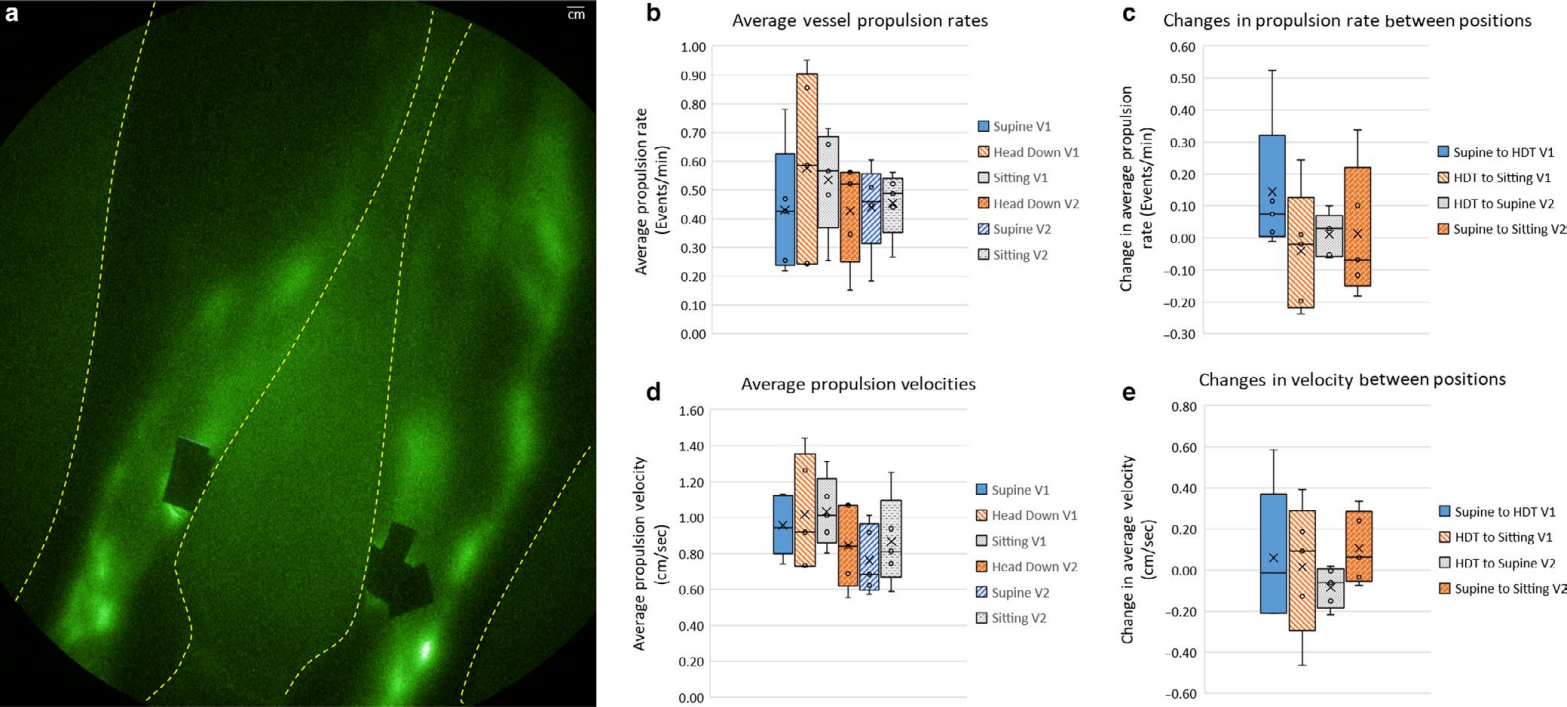
(a) Typical example of lymphatic imaging of left and right lower extremities with Supplemental videos 1, 2, and 3 showing lymphatic contractile activity under HDT, supine, and sitting positions. (b) Whisker plot of the average propulsion rate (min^−1^) as a function of position in Visits 1 and 2. (c) Whisker plot of the change in average propulsion rate (min^−1^) as a function of position in Visits 1 and 2. (d) Whisker plot of the average propulsion velocity (cm/sec) as a function of position in Visits 1 and 2. (e) Whisker plot of the change in average propulsion velocity (cm/sec) as a function of position in Visits 1 and 2. In the boxes, the x denotes the average and the line denotes the median

Figure 7a illustrates a typical example of the lymphatics of the lower extremities and average vessel propulsion rates and velocity in each position within each visit. Lymphatic vessels in the extremities fill nearly instantaneously upon injection, hence we monitored lymphatic pumping as a function of position. Supplemental Videos 1, 2, and 3 show an example of the changing lymphatic contractile activity in the supine (not impacted by gravity), HDT (aided by gravity), and sitting (opposed by gravity) positions. Figure 7b and 7c provide an analysis of all five study subjects showing that average propulsion rates increased in Visit 1 when changed from the gravity neutral supine position to the HDT position and tended to decrease when changed from HDT to sitting. In Visit 2, there was minimal change in propulsion rates when moved from HDT to supine and when moved from supine to sitting position, the contractile activity increased in three of five subjects. The increase in contractile activity moving from supine to HDT positions at Visit 1 was observed in four of five subjects but was not found to be statistically significant. Figure 7d and 7e provide an analysis of all five study subjects showing that the average velocity in the HDT position at both Visits 1 and 2 was higher than the supine position. The velocities in the sitting positions were also higher than in the supine position and were similar to those in the HDT position; however, no statistical significance was found between the velocities at any position in these pilot studies.

## 4 DISCUSSION AND CONCLUSIONS

Facilitating CSF outflow into the lymphatic system may prove critical in the management of SANS under microgravity conditions, but may also play a role in intracranial pressure disorders, and possibly in some neurodegenerative, neuroinflammatory, and psychiatric disorders. Medical imaging of CSF movement has recent, but limited success. Intrathecal iodinated CT or gadolinium MR cisternography provide exquisite images of pathologic CSF leaks and shunts (Holbrook & Saindane, 2017), and phase‐contrast MR and time‐spatial labeling has rapidly evolved to enable visualization of CSF movement in the CNS (Yamada et al., 2015), but there have been limited studies to image CSF outflow into the lymphatics; itself an area of recent controversy and rediscovery (An et al., 2015). Our previous work in mice showed the ability to conduct noninvasive NIRF imaging of CSF drainage and transit time from the subarachnoid space to the draining lymph nodes after intrathecal injection of ICG in WT and Alzheimer's (AD) transgenic mice (Kwon et al., 2019). Others injected dyes and tracers into the cisterna magna of WT and AD mice to show impaired CSF outflow into the cervical LNs (Peng et al., 2016). In the larger swine model, NIRF imaging was complicated by the reflective nature of swine skin and the deep location of the structures, but did confirm that ICG injected intrathecally in the lumbar spine transited to the cranial vault and drained across the cribriform pate into the nasal mucosa. At post‐mortem exam, the ICG was traced from the nasal mucosa to the lymphatic duct adjacent the facial vein, to the lymphatics adjacent the jugular vein, and was seen to accumulate in the associated lymph nodes. While ICG is contraindicated for intrathecal injection in humans, we rationalize that by visualizing lymphatic drainage from the palatine tonsil, the drainage pathway of CSF through the head and neck region could likewise be visualized.

Previously, we used NIRF lymphatic imaging to longitudinally evaluate deep internal lymphatic drainage in head and neck cancer survivors who experienced LN dissection as part of their cancer treatment (Rasmussen, Tan, et al., 2017). Injections in the palatine tonsils were chosen to demark the deep lymphatic drainage pathways that were, more often than not, undetected using the technique, presumably because of the history of cancer and its treatment, inconsistency of the injection depth, or the relatively short imaging time post injection. Herein, in healthy normal subjects with intact deep lymphatic pathways, we routinely visualized the draining lymph nodes and lymphatic vessels from ICG injection in the palatine tonsils. Because injections were made in the sitting position, drainage commenced immediately and, because we employed a stable working standard to enable imaging comparisons across time and study subjects, we could compare across visits and positions. However, in this pilot study, additional CSF drainage occurred during breaks between HDT and sitting or supine positions in which subjects were permitted to stand and walk to the bathroom. Without enforcing supine positioning throughout the study, we cannot rule out that the increase in drainage to the cervical LNs over time was independent of position. Nonetheless, with the data presented herein, future studies that focus upon interventions, the influence of sleep (Xie et al., 2013), novel pneumatic compression (Gutierrez et al., 2019), or postural changes that might impact CSF outflow could be properly designed and powered to eliminate these artifacts. Furthermore, while ICG administration in the palatine tonsils appeared to provide a consistent measure of drainage to the LNs, CSF outflow, and transit times to draining LNs may be more accurately and noninvasively evaluated with NIRFLI following intrathecal administration as demonstrated in rodent models (Kwon et al., 2017).

In addition to evaluating CSF outflow, our study attempted to determine whether peripheral contractile lymphatic function in the lower extremities contributed to cephalad shift. An early work by Olszewski (2008) suggested that in healthy individuals, lymphatic pumping is insensitive to lower extremity position and muscular activity. But compared to the circulatory system, there is a paucity of information on the determinants of lymphatic function. Like the venous circulation, healthy lymphatics possess valves which prevent backflow or “reflux,” but unlike the venous circulation, the lymphatic vessel segments bounded by these values have smooth muscle contractility that dynamically pump lymph in manners that do not appear to be governed by breathing, heart rate, or surrounding muscle motion (Zawieja, 2009). In bipeds, lymph is typically pumped against gravity when standing or sitting. Under normal physiologic conditions, lymphatic contractile activity is thought to be caused in part by the passive or active filling of lymphangions that lead to increased circumferential stresses, transmural pressure, and stimulation of the smooth muscle contraction. This suggests that contractile activity should increase when moving from supine to sitting positions (as shown in the trends depicted in Figure 7b through 7e), wherein gravity aids the filling and distension of *distal* lymphangions to result in increased contractile activity in the lower extremities. Because standing and sitting translocates a large fraction of blood volume to the lower extremities resulting in microvascular filtration from plasma to the interstitium that reduces blood volume, lymphatic pumping helps to restore blood volume (Huxley & Scallan, 2011) while standing or sitting. Given that sympathetic and parasympathetic nerve systems have been shown in humans, (Mignini, Sabbatini, Coppola, & Cavallotti, 2012) autonomic control through the arterial baroflex system may be responsible for mobilizing lymphatic pumping in the lower extremities (Hedrick, McNew, & Crossley, 2015). Not surprisingly our data also showed that contractile activity in the lower extremities increased upon moving from supine to HDT positions, in contrast to the animal work on perfused lymphangions by others (Bulekbaeva, 1989; Gashev, Delp, & Zawieja, 2006). Whether gravity induced‐filling of *proximal* lymphangions caused stimulation of contractile activity or whether other mechanisms are responsible for stimulation of lymphangion activity remains to be investigated. Nonetheless, under HDT, increased lymphatic contractile activity, in both propulsion and velocity, in the lower extremities may contribute to cephalad‐shifts. However, our data suggest that without gravitational forces opposing or enhancing the direction of lymphatic flow in the lower extremities, contractile lymphatic activity alone may not play a role in causing cephalad shift. Indeed, in the gravity neutral supine position, average lymphatic propulsion rates and velocities tended to be lower. Although diminished fluid volume in the lower extremities is reported under microgravity conditions due to reduced venous blood pooling, it is likely that the lower extremity lymphatics do not play a direct role in SANS.

In contrast to our measurements of lymphatic drainage into the cervical LNs, the assessment of lymphatic contractility in the lower extremities is not dependent upon past dye accumulation and therefore may not be as impacted by the study “breaks” as the intensity measurements in the cervical area. Variability in lymphatic contractile activity that was comparable to past studies (Tan et al., 2011), prevented us from detecting statistically significant changes with change in position in this pilot study. Nonetheless, we found average propulsion rates and velocities tended to be higher in HDT and sitting positions when compared to the gravity‐neutral supine position.

Extracranially, lymphatic function is essential for clearance of waste products and inflammatory mediators from tissues, as well as for the delivery of antigens for either inducing tolerance or stimulating adaptive immune response to mediate the immune status of draining tissues. Impairment of peripheral lymphatics results not only in lymphedema, but also in inflammation in draining tissues and impaired immune responses. Whether impaired CSF drainage into the peripheral lymphatics increases ICP and mediates neuroinflammation in humans remains to be fully investigated not only in SANS, but other syndromes and chronic conditions in which impaired lymphatic/CSF drainage restricts clearance of protein and waste products. Methods to stimulate drainage in the CSF drainage pathways could include pneumatic compression devices (Gutierrez et al., 2019) or manual lymphatic drainage techniques. While pro‐inflammatory cytokines inhibit lymphatic function and drainage (Aldrich & Sevick‐Muraca, 2013; Aldrich et al., 2017; Scallan, Zawieja, Castorena‐Gonzalez, & Davis, 2016), till date there are limited studies on how to pharmacologically stimulate lymphatic drainage, particularly those draining the intracranial space. Finally, while mucosal injection into the palatine tonsils may provide convenient access to the drainage pathway shared by CSF outflow, it remains to be validated as a robust diagnostic. The reformulation of ICG to include albumin to prevent precipitation has been previously used in humans (Hutteman et al., 2011). While such a formulation may eliminate concerns of associated neurotoxicity with injection into the CSF, safety and toxicity studies remain to be performed for the intrathecal route of administration.

## CONFLICT OF INTERESTS

JCR, EMS‐M, and CEF are listed as inventors on patents related to near‐infrared fluorescence lymphatic imaging and may receive future financial benefit from its commercialization. JCR, EMS‐M, CEF and The University of Texas Health Science Center at Houston have research‐related financial interests in Lymphatics Technologies, Inc. The remaining authors have no financial relationships relevant to this article to disclose.

## AUTHOR CONTRIBUTIONS

EMS and CEF conceived of experiments; RJK, JCR, SK, JRM, and AP conducted and analyzed the human studies; SK, JCR, FCV, AB, and CFJ conducted and analyzed the animal studies; JCR, SK, CEF, CFJ, and EMS wrote the manuscript, all authors critically reviewed manuscript.
